# Bouveret syndrome and its imaging diagnosis

**DOI:** 10.1590/0100-3984.2016.0220

**Published:** 2018

**Authors:** Isabela Lemos Murelli Rodrigues

**Affiliations:** 1 Conjunto Hospitalar do Mandaqui, São Paulo, SP, Brazil

**Figure 1:**
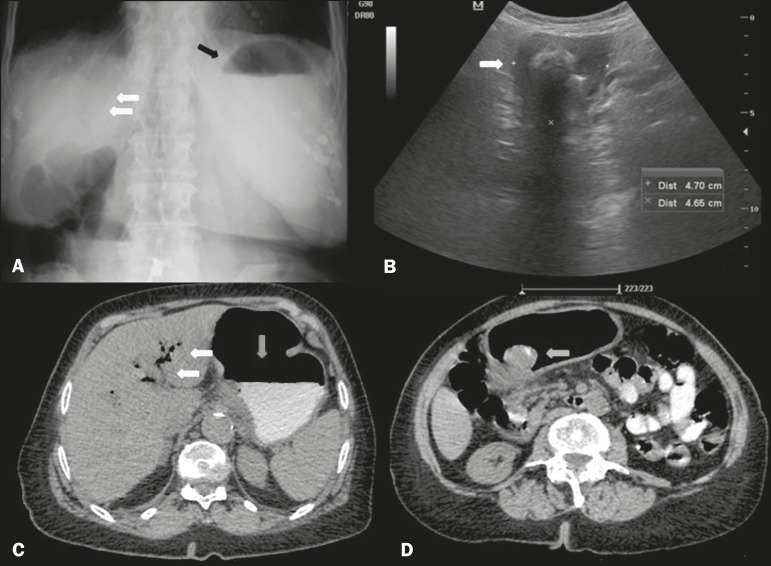
**A:** X-ray of the abdomen, with the patient standing, showing an air-fluid level in the stomach (black arrow) and intrahepatic pneumobilia (white arrows). **B:** Abdominal ultrasound showing the pyloricduodenal region with a calculus impacted in its interior (arrow). **C:** Oral contrast-enhanced computed tomography of the abdomen, showing an air-fluid level in the stomach (vertical arrow) and gaseous content in intrahepatic biliary tracts/pneumobilia (horizontal arrows). D: Oral contrast-enhanced computed tomography of the abdomen, showing a mixed-density calculus in the pyloric region (arrow), causing upstream obstruction and dilation (i.e., of the stomach).

Dear Editor,

An 84-year-old female patient with hypertension reported pain in the upper abdomen accompanied by immediate postprandial nausea and vomiting, without gas or stool elimination for three days. The physical examination showed that she was afebrile, with a distended abdomen, pain upon deep palpation of the upper abdomen, and no signs of peritoneal irritation. A conventional X-ray of the abdomen (Figure 1A) showed air in a branched configuration in the hepatic projection and an air-fluid level in the gastric chamber. Ultrasound (Figure 1B) showed intrahepatic and extrahepatic bile ducts of normal size and the presence of pneumobilia, with no gallbladder identified. There was distension of the gastric chamber, the distal segment appearing to be adhered to the hepatic hilum, as well as a calculus in the pyloric antrum, suggesting the diagnostic hypothesis of gastric obstruction by a gallstone. For better diagnostic elucidation and evaluation of possible complications, we performed computed tomography of the abdomen (Figures 1C and 1D), which demonstrated a correlation with the ultrasound findings, confirming the imaging diagnosis of Bouveret syndrome.

Bouveret syndrome is a rare cause of gastric outlet obstruction due to large-scale impaction of a large gallstone in the duodenal bulb/pylorus after migration through a cholecystoduodenal/cholecystogastric fistula^(1)^. Gallstone ileus is a disease that mainly affects women, and the pathophysiology is often explained by a previous episode of acute cholecystitis^(2)^. The incidence is highest in elderly individuals with comorbidities or biliary tract diseases. The following distribution of gallstone ileus sites has been described^(1,3)^: terminal ileum, 60%; proximal ileum, 24%; distal jejunum, 9%; colon and rectum, 2-4%; distal duodenum, 1-3%; and, less frequently, the proximal portion of the duodenum, where it causes immediate obstruction of emptying. Of all cases of gallstone ileus, 1-3% result from impacted stones in the pyloric or duodenal region, a condition known as Bouveret syndrome.

The diagnosis of Bouveret syndrome can be suspected on the basis of conventional X-ray findings, especially Rigler's triad (Rigler's sign), which is pathognomonic of gallstone ileus and appears in 40-50% of the cases in which conventional X-ray is employed. Rigler's triad is the combination of dilated loops with an air-fluid level, ectopic biliary lithiasis, and gas in the biliary tract^(4)^. Contrast-enhanced imaging of the upper digestive tract may be useful, with visualization of a filling defect, corresponding to the gallstone, and contrast enhancement of the orifice of the cholecystoduodenal/cholecystogastric fistula^(3,5)^. Ultrasound can show pneumobilia, gastric distension, and dilation of intestinal loops, as well as sometimes showing gallstones. Rigler's triad is most commonly seen on tomography scans, on which aerobic and gastric chamber dilatation are easily identified and the fistula can be diagnosed after administration of oral contrast, characterizing its leakage, or indirectly by the identification of contrast enhancement within the gallbladder^(3)^. Although prompt diagnosis can promote the rapid extraction of a gallstone, mortality remains relatively high, especially among elderly patients and patients with comorbidities, because the extraction requires surgical intervention^(6,7)^.

Although Bouveret syndrome is a relatively rare disease, the diagnosis can be made on the basis of the imaging findings, thus allowing early endoscopic and surgical intervention^(1-8)^.
